# Functional Cure of Hepatitis B Virus Infection in Individuals With HIV-Coinfection: A Literature Review

**DOI:** 10.3390/v13071341

**Published:** 2021-07-11

**Authors:** Anders Boyd, Lorenza N. C. Dezanet, Karine Lacombe

**Affiliations:** 1 Department of Infectious Diseases, Research and Prevention, Public Health Service of Amsterdam, 1018 WT Amsterdam, The Netherlands; 2 Stichting HIV Monitoring, 1105 BD Amsterdam, The Netherlands; 3 Institut Pierre Louis d’Épidémiologie et de Santé Publique, INSERM, IPLESP, Sorbonne Université, 75012 Paris, France; lorenza.dezanet@iplesp.upmc.fr (L.N.C.D.); karine.lacombe2@aphp.fr (K.L.); 4 APHP, Hôpital Saint-Antoine, Service de Maladies Infectieuses et Tropicales, 75012 Paris, France

**Keywords:** HBsAg seroclearance, HBsAg seroconversion, nucleoside/nucleotide analogue, chronic hepatitis B, human immunodeficiency virus

## Abstract

In individuals infected with hepatitis B virus (HBV), the loss of hepatitis B surface antigen (HBsAg) is the ultimate therapeutic goal, which defines “functional cure.” For individuals living with human immunodeficiency virus (HIV), functional cure occurs roughly 2 per 100 person-years during potent anti-HBV containing antiretroviral therapy. Although this rate may be higher than expected in treated HBV mono-infected individuals, rates of functional cure widely vary between studies (0.6–10.5 per 100 person-years). Similar to HBV mono-infection, the phase of HBV infection, HBV (sub-)genotypes and hepatitis B “e” Ag-negative variants are associated with functional cure in treated HIV-HBV co-infection. In specifically HIV-HBV co-infected individuals, strong increases in CD4+ T cell counts after treatment initiation have also been linked to functional cure, yet this finding is inconsistent across studies. Several markers directly or indirectly reflecting HBV activity are being developed to predict functional cure, such as quantification of HBsAg, hepatitis B core-related antigen, HBsAg protein composition, anti-hepatitis B core antibodies and interferon-gamma-inducible protein 10. Few have been assessed during treatment in HIV-HBV co-infected individuals and none have been validated to predict functional cure. Novel therapeutics for HBV cure are essential for individuals with HIV-HBV co-infection and need to be separately evaluated in this population.

## 1. Introduction

In people living with the human immunodeficiency virus (HIV) worldwide, roughly 8.4%, or 3.2 million, are thought to be co-infected with chronic hepatitis B virus (HBV) infection [1]. Without effective treatment against HBV, the risk of hepatocellular carcinoma (HCC), liver-related and overall death are increased in HIV-positive individuals with HBV co-infection [2,3]. The first nucleoside/nucleotide analogue (NA) available with dual antiviral activity against both HIV and HBV replication was lamivudine (LAM). The major drawback of this agent is that HBV strains with LAM resistance rapidly emerge during treatment [4], although LAM-resistance occurs less frequently when HBV DNA and transaminases levels are low [5,6,7]. The NA, tenofovir, and its prodrugs tenofovir disoproxil (TDF) and tenofovir alafenamide (TAF) demonstrate more potent anti-HIV and anti-HBV activity with the added advantage of the virtually null risk of developing TDF-resistant HBV variants [8,9]. TDF or TAF is therefore an ideal agent to include as part of antiretroviral therapy (ART) for HIV-HBV co-infected individuals. Accordingly, every major therapeutic guideline recommends treating co-infected individuals with TDF-/TAF-containing ART.

Despite the optimal HBV DNA suppression offered by TDF-containing ART, HIV-HBV co-infected individuals do not appear to exhibit the same regression of liver fibrosis as observed in TDF-treated HBV mono-infected individuals [10,11,12] and a small proportion of co-infected individuals still exhibit HCC [3]. These data contend that virological response might not be an adequate marker for improved prognosis and other criteria are needed to assess therapeutic success more adequately.

Indeed, there are several important serological endpoints that are considered important milestones during HBV infection [13]. For individuals with hepatitis B “e” antigen (HBeAg), the loss of HBeAg, which is termed “HBeAg-seroclearance”, confers reduced incidence of HCC and liver-related death so long as transaminase and HBV replication are reduced. For all chronically infected individuals, the loss of hepatitis B surface antigen (HBsAg), which is termed “HBsAg-seroclearance”, usually represents clearance of HBV infection. Of course, the ability to detect HBsAg loss depends on the assay detection limit and ability to detect HBsAg variants, particularly when HBsAg levels are low [14]. Since HBsAg-seroclearance is commonly observed with undetectable HBV DNA in serum and is associated with a very low risk of developing HCC and improved survival, it is an ideal endpoint to define “functional cure” [15]. Functional cure in the context of HBV mono-infection is considered as HBsAg-seroclearance in the absence of treatment. Considering HIV-HBV co-infected patients are highly discouraged to interrupt their treatment regimen [16], functional cure during co-infection is usually based on HBsAg-seroclearance.

Over the past decade, a wide body evidence has examined the rates of functional cure in HIV-HBV co-infected individuals during anti-HBV containing ART. These studies have found several important determinants of functional cure that could be useful for further study. Considering the importance of functional cure, there have been attempts at finding biomarkers that could assist clinicians in more accurately predicting HBsAg-seroclearance and that have been recently examined in HIV-HBV co-infected individuals undergoing long-term treatment with TDF. The aim of this literature review is then to summarize these aspects of HBV functional cure in the context of treated HIV-HBV co-infection.

## 2. Functional Cure in HIV-HBV Co-Infected Individuals Undergoing Potent Anti-HBV Therapy

Understanding functional cure in the HIV-HBV co-infected population requires some knowledge of how frequently HBeAg- or, more importantly, HBsAg-seroclearance occurs during treatment. Both HBeAg- and HBsAg-seroclearance involve a large immunological component. Given the immunosuppression associated with HIV infection, there is debate as to whether these rates differ between HIV-HBV co-infected versus HBV mono-infected individuals. In this section, we summarize seroclearance rates in individuals with HIV-HBV co-infection and compare them to individuals with HBV mono-infection. The selection of studies is described in Table 1.

### 2.1. Rates of HBeAg- and HBsAg-Seroclearance in HIV-HBV Co-Infected Individuals

**Figure 1:**
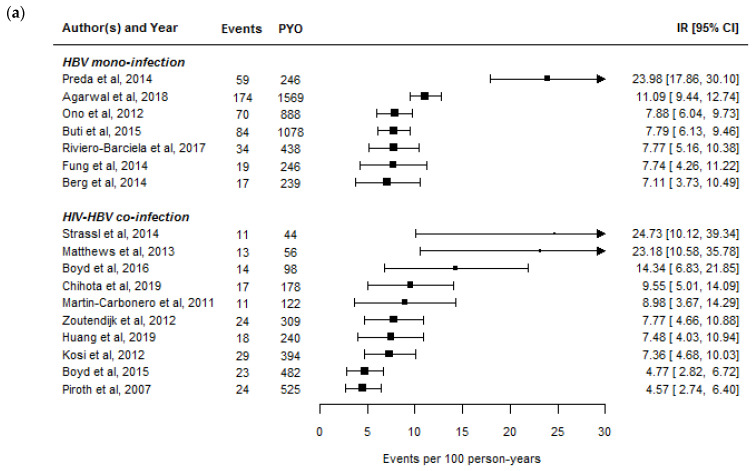

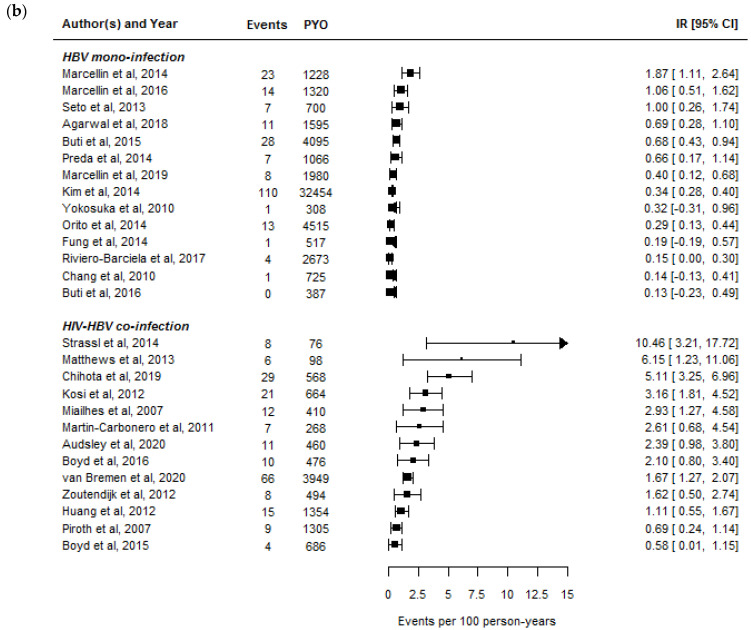
Summary of hepatitis B “e” antigen seroclearance (**a**) and hepatitis B surface antigen seroclearance rates (**b**) in studies of hepatitis B virus (HBV) mono-infected and human immunodeficiency virus (HIV)-HBV co-infected individuals undergoing potent anti-HBV therapy with nucleoside/nucleotide analogues. The size of the squares is proportional to the number of study participants. The selection of articles is described in Table 1. The assumptions used to derive seroclearance estimates are described in the Supplementary Methods S1.

Several studies have examined HBeAg-seroclearance rates in HBeAg-positive co-infected individuals who were either exclusively or almost all treated with ART containing tenofovir and/or LAM [17,18,19,20,21,22,23,24,25]. These rates are summarized in Figure 1a. Overall, HBeAg-seroclearance occurs at a median rate of 8.4 per 100 person-years and within 5 years of therapy, approximately half of HBeAg-positive individuals should be able to achieve HBeAg-seroclearance. Long-term studies would also suggest that these rates are largely unchanged after 15 years of therapy [26]. Acquiring anti-hepatitis B “e” (anti-HBe) antibodies along with HBeAg-seroclearance, which defines “HBeAg-seroconversion,” is expected to occur at a median rate of 4.1 per 100 person-years [17,18,19,21,22,23,24,25,27]. Nevertheless, anti-HBe antibody serostatus commonly fluctuates between positive and negative status in co-infected individuals, suggesting that HBeAg-seroconversion is not a stable event.

These studies and others have also evaluated HBsAg-seroclearance rates during ART containing an anti-HBV agent [17,18,19,20,21,22,23,24,25,27,28,29,30]. These rates are summarized in Figure 1b. HBsAg-seroclearance occurs at a fairly low median rate of 2.39 per 100 person-years and within 5 years of therapy, 8–10% of individuals lose their HBsAg-positive status. The median rate of treated co-infected individuals able to achieve HBsAg-seroclearance and acquire anti-hepatitis B surface (anti-HBs) antibodies, which defines “HBsAg-seroconversion,” is at 0.92 per 100 person-years [17,18,19,20,21,22,23,24,25,28,30]. In general, HBsAg-seroclearance will eventually lead to HBsAg-seroconversion in roughly half of co-infected individuals; however, anti-HBs antibodies can take years to develop and when present, are at relatively lower levels than expected after acute HBV infection in HIV-negative individuals or after HBV vaccination [31].

### 2.2. Rates of HBeAg- and HBsAg-Seroclearance between HBV Mono-Infected and HIV-HBV Co-Infected Individuals

When examining the recently published rates of seroclearance and seroconversion in HBV mono-infected individuals either exclusively or almost all treated with a potent NA, it appears that rates of HBeAg-seroclearance [32,33,34,35,36,37,38] and HBeAg-seroconversion [32,33,34,35,36,37,38,39,40,41,42] are comparable, and rates of HBsAg-seroclearance [32,33,34,36,37,40,41,42,43,44,45,46,47] and HBsAg-seroconversion [32,34,36,38,41,43,46] lower compared to treated HIV-HBV co-infected individuals (Table 2). For both infection groups, HBsAg-seroclearance occurs infrequently.

Although the range in HBeAg-seroclearance rates between studies is comparable between HBV mono-infected and HIV-HBV co-infected individuals (Figure 1a), the range in HBsAg-seroclearance is much wider in HIV-HBV co-infected than HBV mono-infected individuals. Part of the reason could be due to differences in study populations as HBV mono-infected cohorts generally have a higher proportion of individuals with HBeAg- negative serology, for whom HBsAg-seroclearance is known to occur at a much lower rate [13]. In addition, HBV cohorts more often have regularly collected serological data than large cohorts of HIV-positive individuals with chronic HBV infection, and the large gaps in HBeAg and HBsAg testing could bias rates to be lower. However, several studies in

HIV-HBV co-infected individuals have shown a remarkably fast rate of HBsAg-seroclearance within a very short period after initiating anti-HBV containing ART [19,23,25]. The differences between studies highlight that some subpopulations would appear to have an advantage with respect to functional cure.

## 3. Determinants of Functional Cure during Treated HIV-HBV Co-Infection

Past research in HBV mono-infected individuals has uncovered a wide range of determinants associated with functional cure during treatment. These include the specific phase of HBV infection, HBV (sub-)genotypes, HBeAg-negative variants, and genetic characteristics of the host. For individuals with HIV-HBV co-infection, this list extends to HIV-related characteristics, such as CD4+ T-cell counts and acquired immunodeficiency syndrome (AIDS)-defining illnesses. In this section, we summarize how the determinants of functional cure in HBV mono-infection relate to HIV-HBV co-infection, while devoting particular attention to the effects of immunosuppression.

### 3.1. Phase of HBV Infection

Lower baseline HBsAg levels and lower baseline HBV DNA levels have been associated with increased rates of HBsAg-seroclearance in both HBV mono-infected individuals in general [48] and HIV-HBV co-infected individuals initiating anti-HBV containing ART [25,27,49]. The lower levels of these markers are correlated with duration of chronic HBV infection [13], which suggests that the timing of HBV acquisition plays an important role in functional cure. In settings where HBV is endemic (with vertical transmission during birth occurring more frequently in Asia and horizonal transmission between children/youth occurring in Africa), HIV is commonly acquired well after HBV infection, whereas in settings where HBV is non-endemic (with sexual transmission or injecting drug use in Europe, Australia, and North America), HIV and HBV acquisition are commonly acquired at the same time [50]. Accordingly, a large study of treated HIV-HBV co-infected individuals has demonstrated that those of African origin have a much higher probability of being able to clear HBsAg than Caucasians [51]. Older age has also been commonly found to be associated with HBsAg-seroclearance in treated co-infected individuals [52], which might also reflect individuals with longer duration of HBV infection.

### 3.2. HBV (Sub-)Genotypes and HBeAg-Negative Variants

Certain virological factors of the HBV genome have also been associated with functional cure during therapy with NAs [53]. When harboring mutations on the *precore* gene, even existing as a minority quasi-species, rates of HBsAg-seroclearance are reduced during treatment [54]. Similar results were observed in HIV-HBV co-infected individuals initiating anti-HBV containing ART in which HBsAg-seroclearance was only observed in those who did not harbor HBV strains with the G-to-A substitution of the 1896 nucleotide on the *precore* gene [55]. *Precore* mutations of these kind tend to appear with longer duration of HBV infection [56], suggesting that earlier treatment before these mutations develop might increase the probability of HBsAg-seroclearance. Notably, HBV genotype is closely linked to the presence of *precore* mutations [57] and the geographic differences in HBV (sub-)genotype distribution (i.e., A1 or E in Sub-Saharan Africa, B or C in Asia [58]) could explain regional variation in the rates of HBsAg-seroclearance.

The “a” determinant of the major hydrophilic region on the *S*-gene is a major target for neutralizing antibodies, hence mutations in this region have been shown to influence antigenicity and possibly infect vaccinated hosts [59]. It could be hypothesized that mutations of this sort would also influence the HBsAg-seroclerance. Indeed, several studies have demonstrated that decreased genetic complexity, less frequent or specific mutations, and less frequent deletions in the “a” determinant are correlated with clearing HBsAg [60,61,62]. One study has examined the broader spectrum of *S*-gene mutations in co-infected individuals prior to initiating anti-HBV containing ART, including mutations on the “a” determinant, and failed to find any mutations linked to HBsAg-seroclearance [63]. Hence, *S*-gene mutations might not affect rates of HBsAg-seroclearance during treatment.

### 3.3. Genetic Variability of the Host

Several studies have found that genetic variants on the human leucocyte antigen (HLA)-DP gene are associated with HBsAg-seroclearance during the natural history of infection [64] or during treatment with NA [65], which seems to be consistent across different populations [66]. Other polymorphisms on genes linked to immunological function, such as the toll-like receptor 3 [67], interleukin 10 [68], and T-cell immunoglobulin and mucin domain-containing molecule 3 genes [69], have also shown to be linked to the natural progression of HBV infection and are less consistently studied [66]. There have been very few studies examining the role of genetic polymorphisms on HBsAg-seroclearance in HIV-HBV co-infected individuals, apart from the IL28B haplotype showing no association [70]. Another research group found an association with HLA-G 14-bp insertion and deletion polymorphisms with HBsAg-seroclearance in HBV mono-infected, but not HIV-HBV co-infected, individuals [71]. These results would suggest that any effect of genetic polymorphisms on functional cure could be overshadowed by other host factors in co-infected individuals, namely immunosuppression.

### 3.4. Immunosuppression

It was first noted in the late 2000s that some HIV-HBV co-infected individuals with AIDS-defining illnesses were able to rapidly clear HBeAg/HBsAg shortly after initiating ART. Other studies have also recently shown that higher increases in CD4+ T-cell count during the first years of ART or initiating ART with lower CD4+ T-cell counts, and not necessarily AIDS, were associated with HBsAg-seroclearance [20,30]. Interestingly, many of the studies listed in Figure 1b with noticeably higher HBsAg-seroclerance rates were conducted in individuals with extremely low CD4+ counts who initiated ART. These results together would suggest that an immune reconstitution inflammatory syndrome (IRIS)-type effect is occurring and that the rapid expansion of T-cells in combination with specific inflammatory markers could help usher HBsAg-seroclearance [72]. It remains unknown, however, which specific pathways are responsible for eliciting HBsAg-seroclearance and if known, could provide important information on the potential targets for functional cure.

During HBV mono-infection, HBsAg itself is known to induce a wide range of effects on immune cells (reviewed in [73]). As HBsAg levels decrease, exhausted innate immunity is restored and capacity to activate immune cells involved in both cytolytic and non-cytolytic activity, as well as cells involved in producing neutralizing anti-HBsAg antibodies, is improved. However, when compared to HBV mono-infection, most of these cell populations are much lower in the liver microenvironment of individuals with HIV-HBV co-infection and after initiating anti-HBV containing ART, do not substantially increase over time [74]. These data illustrate that HBsAg-seroclearance may be even impaired in some HIV-HBV co-infected individuals due to poor anti-HBV specific responses. If therapeutic agents targeting immunological pathways are successful in HBV mono-infected individuals, it will need to be determined whether the immune impairments observed in co-infected individuals affects their therapeutic efficacy.

## 4. Surrogate Markers of HBV Functional Cure in HIV-HBV Co-Infection

The generally accepted marker to evaluate active HBV replication is serum HBV DNA. However, HBV DNA is unable to accurately predict functional cure. There has been a substantial amount of research attempting to find other markers that could bear a higher capacity to predict HBsAg-seroclearance [75]. Many of these new markers are by-products of the HBV replication cycle, secreted from the hepatocytes into circulating blood [76]. These markers *directly* reflect HBV activity. Other markers have relied on components of the host immune system, such as markers of inflammation or antibody production against the virus. These markers *indirectly* reflect HBV activity. Both direct and indirect markers have been recently validated in HBV mono-infected individuals to predict disease phase, and viral replication as well as more severe clinical endpoints, such as levels of liver fibrosis and HCC. In this section, we focus on the use of these markers in treated HBV mono-infected individuals compared to treated HIV-HBV co-infected individuals with respect to HBeAg- and HBsAg-seroclearance (Table 3).

### 4.1. Direct Markers of HBV Activity

#### 4.1.1. Quantification of Hepatitis B Surface Antigen

HBsAg is translated from pre-S1 and S2 messenger RNAs on covalently-closed circular (ccc) HBV DNA (i.e., the viral template needed to express HBV proteins), which are transcribed from the *S* gene [75]. However, HBsAg can also be produced from integrated HBV DNA. Quantified HBsAg (qHBsAg) levels strongly correlate with ccc-DNA [77] and hence could be used as a potential marker to reflect HBV activity. This activity is likely to wane during antiviral treatment. Accordingly, the capacity of qHBsAg levels to predict serological responses were extensively studied in the early 2010s and when determined at baseline and one-year later, qHBsAg levels were able to rather modestly predict HBeAg- and HBsAg-seroclearance during treatment with pegylated interferon (peg-IFN) [78]. Nevertheless, the major issue with qHBsAg is that levels oftentimes stagnate during prolonged therapy, making it difficult to relate to other replicative parameters (i.e., intrahepatic replication [79]) and clinical outcomes in general [80]. For these reasons, no guidelines recommend its use in routine practice [14].

The use of qHBsAg has been examined during TDF-containing ART [18,28] in HIV-HBV co-infected individuals, and declines in this marker would appear to be comparable to HBV mono-infected individuals on NA. Given the few events in these studies, it was not possible to establish baseline levels or decline in levels predictive of HBsAg-seroclearance. Nevertheless, a group-trajectory modeling approach was used to identify specific profiles of qHBsAg during TDF treatment, demonstrating that HBsAg-seroclearance was linked to substantial and immediate declines in qHBsAg [18].

#### 4.1.2. Quantification of Hepatitis B Surface Antigen Protein Composition

HBsAg is composed of large (L), middle (M), and small (S) hepatitis B surface (HBs) cocarboxyterminal glycoproteins that are encoded within the S open reading frame [81]. Recently, it has been found that the proportions of LHBs and MHBs proteins are much lower in individuals with inactive HBV infection and importantly, can predict the phase of HBV infection more accurately than total HBsAg [82]. This finding would make it a potential marker for evaluating HBV activity and thus perhaps serological response to antiviral therapy. Indeed, faster declines in the proportion of LHBs and MHBs were predictive of HBsAg-seroclearance in both NA- and pegylated interferon-treated individuals with HBV mono-infection [83]. The capacity of this marker to predict functional cure in HIV-HBV co-infected individuals has yet to be evaluated.

#### 4.1.3. Quantification of Hepatitis B Core-Related Antigen

The hepatitis B core antigen shares a 149-amino acid sequence with HBeAg and when quantified together, is called hepatitis B core-related antigen (HBcrAg). Its quantification appears to be unaffected by the presence of *precore* mutations [84]. Importantly, strong correlations between HBcrAg and intrahepatic ccc-DNA levels have been observed in both HBV mono-infected and HIV-HBV co-infected individuals [85,86]. In terms of on-treatment use, HBcrAg levels have been shown to be a strong predictor of HBeAg- and HBsAg-seroclearance, particularly in HBV mono-infected individuals undergoing treatment with peg-IFN or LAM [84,87]. Another study has suggested that HBcrAg levels at initiation of NA is not independently associated with HBeAg-seroconversion, but higher levels at the time of seroconversion are linked to remission to HBeAg-positive serostatus [88].

Only one study to date has examined both the on-treatment kinetics and predictive capacity of HBcrAg levels on seroclearance in TDF-treated HIV-HBV co-infected individuals [89]. In this study, baseline HBcrAg levels were indeed associated with HBeAg-seroclearance; however, higher sensitivity and specificity were obtained when HBcrAg levels were evaluated at later time-points during TDF therapy (i.e., 24 to 36 months). The low number of HBsAg-seroclearance events precluded any evaluation of this marker on functional cure.

#### 4.1.4. Quantification of HBV RNA

Some virions found in the cytoplasm of the hepatocyte contain pre-genomic (pg) HBV RNA and other forms of RNA (as total HBV RNA), which can be secreted in the blood [76]. Levels of these RNAs, as detected in the serum, have been correlated with other serum markers of HBV replication (i.e., HBV DNA, qHBsAg, and ALT) and are thought to reflect transcriptional activity of ccc-DNA [90,91]. Hence, HBV RNA quantification would be a candidate marker for serological response. The quantification of serum polyadenylated HBV RNA has been shown to more accurately predict HBeAg- and HBsAg-seroconversion than qHBsAg during treatment with peg-IFN and NA in HBeAg-positive HBV mono-infected patients [92,93]. HBV RNA has never been evaluated in the context of co-infection, which poses certain issues. Intrahepatic replication intermediates, including ccc-HBV DNA, are generally higher in patients with more severe levels of past immunosuppression [79]. Given that serum HBV RNA likely originates from these intermediates, their levels could be potentially higher with more severe degrees of immunosuppression, abrogating their predictive capacity.

### 4.2. Indirect Markers of HBV Activity

#### 4.2.1. Quantification of Interferon-Gamma-Inducible Protein 10

Notwithstanding the wide range of cytokines and chemokines known to be involved in the immune response against HBV infection, interferon-gamma-inducible protein 10 (IP-10) has received considerable attention over the past decade. IP-10 is a chemoattractant for T-lymphocytes, monocytes, and natural killer-cells and its levels are associated with circulating markers of HBV activity [94,95]. It was assumed that IP-10 levels could also function as a prognostic tool during treatment. This marker has been able to accurately predict HBeAg- and HBsAg-seroclearance in HBV mono-infected patients treated with either peg-IFN or NA, with some evidence suggesting that is more accurate than qHBsAg in entecavir-treated HBV mono-infected patients after four years of treatment [96,97,98]. Interestingly, this is the only chemokine among a number of serum cytokines [interleukin (IL)-2, IL-3, IL-4, IL-7, IL-9, IL-10, IL-12, IL-15, IL-21, interferon-γ, tumor necrosis factor-α, granulocyte macrophage colony stimulating factor] that was associated with HBsAg-seroclearance in these studies.

IP-10 is intimately linked with HIV infection. HIV-viremia at various stages of both natural and treated infection is strongly and positively correlated with IP-10 [99,100], with evidence suggesting that mainly monocytes and myeloid dendritic cells are responsible for IP-10 production during HIV replication [101]. Only one pilot study to date has looked at serum IP-10 in HIV-HBV co-infected patients after initiating an anti-HBV containing ART regimen, showing substantial declines in this cytokine but no capacity to predict HBeAg-seroconversion [102].

#### 4.2.2. Quantification of Anti-Hepatitis B Core Antibodies

The major drawback of using chemokine or cytokine levels, or any other marker of immune activation (e.g., ALT levels) is that it is unspecific to adaptive anti-HBV immunity. Several research groups have proposed that measuring anti-hepatitis B core (anti-HBc) antibody levels, as reflection of anti-HBc specific immunity, could provide a more specific indication of host immunity against HBV infection [103]. Indeed, this marker has been evaluated with respect to serological response during treatment. Higher levels of anti-HBc antibodies have been shown to predict HBeAg- and HBsAg-seroconversion in a large study population of Asian, HBeAg-positive, HBV mono-infected patients who were treated with either peg-IFN or NA [104].

B cells play a central role in producing anti-HBc antibodies and are known to express higher levels of inhibitory receptors linked to cell exhaustion during HIV-infection [105]. Anti-HBc levels could be consequently attenuated compared to HBV mono-infection, altering the ability of this marker to predict seroclearance. One study in TDF-treated HIV-HBV co-infected individuals has been performed evaluating anti-HBc levels, showing that higher levels of this marker at TDF-treatment initiation were only able to exhibit high specificity in predicting HBeAg-seroclearance [89]. Although this marker did not perform well in predicting HBeAg-seroclearance, its changes during treatment were associated with sudden decreases liver fibrosis levels [106]. No evaluation of this marker has been made on HBsAg-seroclearance in the treated, HIV-HBV co-infected population.

## 5. Discussion

HBsAg-seroclearance rarely occurs in the majority of studies evaluating HIV-HBV co-infected individuals treated with potent anti-HBV containing ART. Individuals with HBV or HIV-HBV infection share several common determinants of higher rates of HBsAg-seroclearance, such as low HBV DNA and HBsAg levels, lack of *precore* mutations, and older age, while some studies suggest a role of CD4+ T-cell counts on functional cure. Many of the novel markers demonstrating some capacity to predict HBsAg-seroclearance seem to perform comparably well in HIV-HBV co-infected populations; however, these markers do not seem to outperform more conventional markers, such as quantified HBsAg levels.

Overall, the low rates of HBsAg-seroclearance stress the need for other therapeutic regimens that will be able to induce functional cure. Some of the more recent therapeutic strategies evaluated during HBV mono-infection have included add-on with peg-IFN after exposure to NA [107,108] or interruption of anti-HBV therapy [109]. No beneficial effect of peg-IFN intensification after TDF initiation has been observed in HIV-HBV co-infected patients [110], while treatment interruptions are generally discouraged due to their associated risk of hepatic flares and increased morbidity [16,111]. Since residual ccc-HBV DNA and integrated HBV DNA remain present in the hepatocyte after loss of HBsAg, discontinuation of anti-HBV treatment, particularly under immune suppression, could reactivate HBV [112] and thus anti-HBV-containing ART might be required despite functional cure. Nevertheless, previous studies on discontinuation have been mostly conducted in patients who were highly immunocompromised (i.e., low nadir CD4+ cell count) and it is unknown if these treatment modalities would be beneficial for patients with high CD4+ cell counts and immediate ART initiation.

There are a number of promising antiviral agents currently being evaluated in mostly phase 1 and 2 trials, which include entry inhibitors, transcriptional control inhibitors, post-transcriptional control inhibitors, capsid assembly modulators, and HBsAg release inhibitors (reviewed in [113]). Some agents are also being developed to directly affect production of ccc-HBV DNA. Although these agents are expected to block various steps of the replication cycle, it has been argued that suppression of replication needs to occur in tandem with improved immune responses against HBV [114]. Given the implications of HIV-induced immunosuppression on the replication and pathogenesis of HBV infection [115], these agents must be separately evaluated in co-infected individuals.

It should also be noted that the studies linking HBsAg-seroclearance to clinical improvement are found almost exclusively in HBV mono-infected individuals. There is an urgent need to quantify the risk of liver-related outcomes in HIV-HBV co-infected individuals with HBsAg-seroclearance and whether there are specific groups who might benefit less from functional cure. The immunosuppression associated with HIV-infection combined with the fact that integrated HBV DNA remains even though HBsAg is not expressed [15], could contribute to HCC and extra-hepatic manifestations of disease. These issues need to be explored in larger cohorts.

Functional cure remains an elusive therapeutic goal for co-infected individuals. Nevertheless, noticeably higher rates of HBsAg-seroclearance have been known to occur in individuals with accelerated increases of CD4+ T-cell counts, possibly denoting an IRIS-like elicitation of sudden anti-HBV immune response. Identifying the mechanisms underlying accelerated HBsAg-seroclearance could help usher in the development of agents able to mimic these anti-HBV responses, which could be pivotal for HBV cure research.

## Figures and Tables

**Table 1:** Search strategy and selection criteria for this literature review.

Review Sections	Search and Selection Strategy
Rates of functional cure	We conducted a literature search using PubMed on 31 March 2021 to retrieve relevant articles in English. We searched using the terms “HBeAg loss”, “HBeAg seroclearance”, “HBeAg seroconversion”, “HBsAg loss”, “HBsAg seroclearance”, “HBsAg seroconversion”, “functional cure”, “tenofovir”, “entecavir”, “nucleoside analogue”, “nucleotide analogue”, and “hepatitis B virus” alone and in combinations to retrieve an initial list of publications. We restricted studies to those including individuals with HBV mono-infection or HIV-HBV co-infection for whom the majority were undergoing treatment with potent NA. We included studies from this search that had results on the number of events, number of individuals included in analysis, and median/mean time or person-years of follow-up.
Determinants of functional cure	We included studies from the search on rates of functional cure that included an analysis describing characteristics of individuals with higher rates of HBsAg-seroclearance. Since no study from this search included data on genetic polymorphisms, we removed the treatment search terms and included the terms “polymorphism”, “allele”, and “genetic mutation” alone and in combinations.
Markers predicting functional cure	We conducted a literature search using PubMed on 31 March 2021 to retrieve relevant articles in English. We used each marker of HBV replication as a search term (i.e., “HBsAg quantification”, “HBsAg composition”, “hepatitis B core-related antigen”, “pre-genomic HBV RNA”, “HBV RNA”, “IP-10”, and “anti-hepatitis B core antibody”). We restricted studies to those including treated individuals with HBV mono-infection and HIV-HBV co-infection.

**Table 2:** Median rates (range) of serological endpoints related to hepatitis B “e” antigen and hepatitis B surface antigen during treatment for chronic hepatitis B virus infection.

Serological Endpoint	HBV Mono-Infected	HIV-HBV Co-Infected
HBeAg-seroclearance ^1^	7.8 (7.1–24.0)	8.4 (4.6–24.7)
HBeAg-seroconversion ^1^	5.7 (2.2–14.3)	4.1 (1.0–23.2)
HBsAg-seroclearance ^2^	0.37 (0–1.06)	2.39 (0.6–10.46)
HBsAg-seroconversion ^2^	0.44 (0–0.92)	0.92 (0.15–7.85)

Median (range) rates are those from the selection of studies described in Table 1. The methods used to derive these estimates are described in Supplementary Methods S1; ^1^ In HBeAg-positive individuals; ^2^ In both HBeAg-positive and HBeAg-negative individuals. Abbreviations: HBeAg, hepatitis B “e” antigen; HBsAg, hepatitis B surface antigen; HBV, hepatitis B virus; HIV, human immunodeficiency virus.

**Table 3:** Serological endpoints evaluated with novel biomarkers in hepatitis B virus mono-infected and human immunodeficiency virus-hepatitis B virus co-infected individuals.

Biomarker	HBV Mono-Infected	HIV-HBV Co-Infected
Direct Markers of Activity		
HBsAg levels	HBeAg-seroclearance, HBsAg-seroclearance	HBeAg-seroclearance, HBsAg-seroclarance (preliminary)
HBsAg protein composition (L, M, S)	HBsAg-seroclearance (preliminary)	None
HBcrAg levels	HBeAg-seroclearance ^1^ HBsAg-seroclearance (preliminary)	HBeAg-seroclearance, None on HBsAg-seroclarance
pgHBV RNA/HBV RNA levels	HBeAg-seroclearance ^2^ HBsAg-seroclearance	None
Indirect markers of activity		
IP-10 levels	HBeAg-seroclearance, HBsAg-seroclearance	None
anti-HBc antibody levels	HBeAg-seroconversion, HBsAg-seroconversion	HBeAg-seroclearance, None on HBsAg-seroclearance

^1^ Not concordant between studies; ^2^ Only examined in studies involving treatment with pegylated-interferon with or without nucleoside/nucleotide analogues. Abbreviations: anti-HBc, anti-hepatitis B core antibody; HBcrAg, hepatitis B core-related antigen; HBeAg, hepatitis B “e” antigen; HBsAg, hepatitis B surface antigen; HBV, hepatitis B virus; HIV, human immunodeficiency virus; IP-10, interferon-gamma-inducible protein 10; pg, pre-genomic.

## Data Availability

All data and statistical code used to produce the figures can be found on the webpage: https://github.com/boyd0094/INSERM_HBV_functional_cure.

## Supplementary Materials

The following are available online at https://www.mdpi.com/article/10.3390/v13071341/s1, Supplementary Methods S1: Determining seroclearance and seroconversion rates for the present literature review.
