# Supplementary material for: Functional Cure of Hepatitis B Virus Infection in Individuals With HIV-Coinfection: A Literature Review
Source: Viruses. 2021 Jul 11;13(7):1341. doi: 10.3390/v13071341 (PMC8309973; doi:10.3390/v13071341)
Supplement: Supplementary file 1 [file viruses-13-01341-s001.zip › viruses-1296387-supplementary.pdf]

Supplementary Methods S1: Determining seroclearance and seroconversion rates for the present literature review.

The literature search resulted in a total of 17 studies for HBeAg-seroclearance, 20 for HBeAg-seroconversion, 27 for HBsAg-seroclearance and 18 for HBsAg-seroconversion. Information from these articles was extracted and entered in Excel by L.D. and verified by A.B. In order to calculate the person-years of observation (for those studies not reporting this value), we multiplied the median or mean follow-up time with the number of individuals included in the study. The approximation assumes that event and censoring rates are constant over time. We illustrated the variability in incidence rates across studies using forest plots generating by the forest() function of the “meta” package in R. This statistical analysis was carried out in R (v3.6.1, Vienna, Austria) and statistical code, along with the databases, can be found at https://github.com/boyd0094/INSERM_HBV_functional_cure.
